# Pigs’ brain responses to stroking after long-term positive human contacts: an fMRI exploratory study

**DOI:** 10.1038/s41598-026-66310-w

**Published:** 2026-08-10

**Authors:** Oceane Schmitt, Yann Serrand, Bibiane Pollak, Giorgio Mattaliano, Nicolas Coquery, Pierre-Antoine Eliat, David Val-Laillet, Jean-Loup Rault

**Affiliations:** 1 Animal Welfare Science Unit, University of Veterinary Medicine Vienna, Vienna, Austria; 2 INRAE, INSERM, Nutrition Metabolisms and Cancer, NuMeCan, University of Rennes, Rennes, France; 3 Clinical Department for Small Animals and Horses, Clinic for Diagnostic Imaging, University of Veterinary Medicine, Vienna, Austria; 4 Department for Companion Animals and Horses, Anaesthesiology and Perioperative Intensive-Care Medicine, University of Veterinary Medicine, Vienna, Austria; 5 CNRS, INSERM, University of Rennes, Biosit UAR 3480 US_S 018, PRISM, 35000 Rennes, France; 6 Present Address: Chair Animal Husbandry, Behaviour and Welfare, Institute of Animal Breeding and Genetics, Justus-Liebig University of Giessen, Giessen, Germany

**Keywords:** BOLD response, Human-animal relationship, Emotions, Cognition, Neuroimaging, Animal welfare, Neuroscience, Zoology

## Abstract

Positive interactions with humans can induce pleasurable experiences in animals, but their underlying neurobiology mechanisms are unknown. We investigated the brain responses (Functional Magnetic Resonance Imaging) to stroking by a human in 20 pigs under general anaesthesia. Ten pigs received positive human contacts over 9 weeks post-weaning (POS) and 10 pigs did not (CTL). Images from CTL pigs showed peaks of activation in the primary somatosensory cortex, caudate nucleus, anterior prefrontal cortex, and dorsal anterior cingulate cortex during stroking. Greater peaks of activation in the anterior prefrontal, ventral anterior cingulate, primary somatosensory and somatosensory association cortices were observed in POS pigs; whereas greater peaks of activation in the amygdala were observed in CTL pigs. Therefore, stroking is perceived by pigs under general anaesthesia, and the brain activations patterns are consistent with neural processing associated with pleasant or affective tactile stimulation.

## Introduction

Human-animal interactions form an inherent part of the daily life of domestic animals and can have profound impact on animal welfare^1,2^. A vast amount of scientific evidence demonstrates that negative human-animal interactions (e.g. loud noises, sudden movements, pushing, hitting and forceful contact) increase the animals’ fear of humans and trigger the stress response in presence of humans in general^3^. More recent studies found that positive human-animal interactions (i.e. involving soft voice, calm movements, gentle strokes and scratches, and letting the animal choose to approach or not) are beneficial to animals^2,4^. For instance, pigs stroked 5 min per day during lactation showed better resilience (*i.e.* reduced stress response) to handling^5^ and social isolation^6^. Beyond reducing fear of humans and increasing productivity^7^, positive interactions can lead to the establishment of a positive human-animal relationship and trigger indicators of positive emotional states in animals (e.g. pigs^8^ and rats^9^). Human-animal interactions often involve gentle tactile contacts (i.e. strokes, scratches) that are generally characterized as positive based on the behavioural responses of the animal, which seeks and maintains the contact and might be in a relaxed or confident state (e.g. closing eyes, exposing belly). To a certain extent, stroking imitates allogrooming, a positive behaviour occurring between preferred social partners, although they have different sensory properties. Indeed, allogrooming consists of gently touching, licking, rubbing, nibbling or sniffing another pig^10,11^ and stroking consists of repeatedly applying the hand on the body of the animal. Domestic pigs naturally seek contact and proximity with humans, and enjoy being stroked^12,13^. However, it is still unclear how these tactile interactions are processed by the animal at the brain level, and which neurocognitive mechanisms involved.

The first brain response to stroking is expected in the somatosensory cortex, which processes tactile stimulations. For instance, tickling rats results in increased firing of cells in the somatosensory cortex^14^. Furthermore, positive tactile interactions are also expected to constitute social-like (e.g. release of oxytocin during or after interactions^15^) and rewarding situations (e.g. heifers pursuing humans for strokes^16^), associated with pleasure (e.g. pigs relaxing while being stroked^17^), and would thus possibly mobilize brain regions involved in these processes.

Reward learning consolidation is well known to involve the striatum (basal ganglia) together with associative and integrative brain structures such as the amygdala, hippocampus, prefrontal cortex, and anterior cingulate cortex^18^. For instance, sucrose administration in pigs, a palatable gustatory stimulus, activated the right putamen and right caudate, but also the (anterior and dorsolateral) prefrontal cortex and the anterior cingulate cortex^19^. However, pleasant stimulations are translated into different neuronal patterns of activation depending on their nature and sensory modality^18^. For instance, access to a social reward (i.e. images of conspecifics) activated the caudate neurons whereas access to a juice reward activated the putamen and ventral striatum neurons in macaques^20^. Pleasant touch resulted in the activation in several areas such as the putamen and caudate^21^, pregenual anterior cingulate cortex^22^, the prefrontal cortex^23^ and the anterior cingulate cortex^24^ in humans. More specifically, pleasantness ratings of touch predicted activation in the anterior cingulate cortex^25^, and stroke lesions in the insula disrupted the perception of affective touch in humans^26^. Furthermore, people being stroked at the optimal speed for eliciting the C-tactile (CT) afferents discharge (3 cm/s) showed a greater Blood Oxygenation Level Dependent (BOLD) response in the posterior insula than people stroked at non-optimal speed (30 cm/s)^27^. Coactivation in the medial prefrontal cortex and the dorsal anterior cingulate cortex with the left insula and amygdala during tactile stimulation (i.e. arm touch) demonstrated the existence of a network of brain regions beyond the insula involved in coding CT-targeted affective touch^28^. Given the similarities between the pig and human skins^29^ (both having tactile C-fibers) and brains^30^, it can be expected to observe a reaction to tactile stimulation similar to those observed in humans.

Being touched is not only about the pleasure experienced, but also about the social (or pseudo-social in the case of human-animal interactions) significance. In humans, brain activity measured using functional near-infrared spectroscopy (fNIRS) revealed that the activity in the prefrontal cortex was higher when petting a dog compared to petting a plush animal, especially after repeated exposures^31^. Neophobic reactions to unknown stimulations, even if they are intended to be positive in nature, are translated in the brain by the activation of brain areas involved in alertness and vigilance. For instance, non-social tactile stimulation in the form of gentle airflow applied on the face elicited a greater response of macaques’ amygdala neurons compared to facial grooming-like finger sweeps by a trusted human^32^. The caudate also seems to be involved in neophobic reactions, as less activation in the caudate was observed after olfactory exposure in naive but not in experimented anaesthetised pigs regarding a specific odour^33^.

Altogether, the literature shows that the patterns of neural activation in response to a tactile stimulation can be observed across species, both in terms of perception and affective appraisal. However, many studies on the perception of pleasant stroking were performed in conscious humans or animals, and it is unsure how well their results would translate to anaesthetised animals.

Previous studies using functional magnetic resonance imaging (fMRI) in pigs showed that their brain still respond to olfactory^33–36^ or gustatory^37^ stimulations of different valence, and that the hedonic responses to food sensory stimuli depended on previous individual exposures and associative conditioning^34,37^. Therefore, the present study investigated the BOLD responses of anaesthetised pigs to stroking on the back by a human, and their modulation based on previous experience. Two main hypotheses were tested: (1) The neural response to stroking can be detected in anaesthetised pigs, (2) Experience with positive contacts with a human affects the neural response to stroking. Two experimental treatments were compared: Pigs that experienced receiving regular positive human contacts (POS) and pigs that did not (control, CTL). We expected to observe a similar response to stroking in the primary somatosensory cortex in both treatments, as a confirmation that the tactile stimulation was perceived and processed by the brain even under general anaesthesia. We expected to observe a greater response in the prefrontal cortex, in the dorsal striatum (caudate and putamen), in the nucleus accumbens, and in the anterior cingulate cortex in POS pigs compared to CTL, due to an expected increased pleasure (striatum), possibly also to specific attention (anterior cingulate cortex) and engagement to it (prefrontal cortex), and a greater activation in the amygdala in CTL pigs compared to POS due to an expected increased salience of novelty or perhaps negative feelings induced by unusual body touch.

## Results

### Brain response to touch

**Fig. 1:**
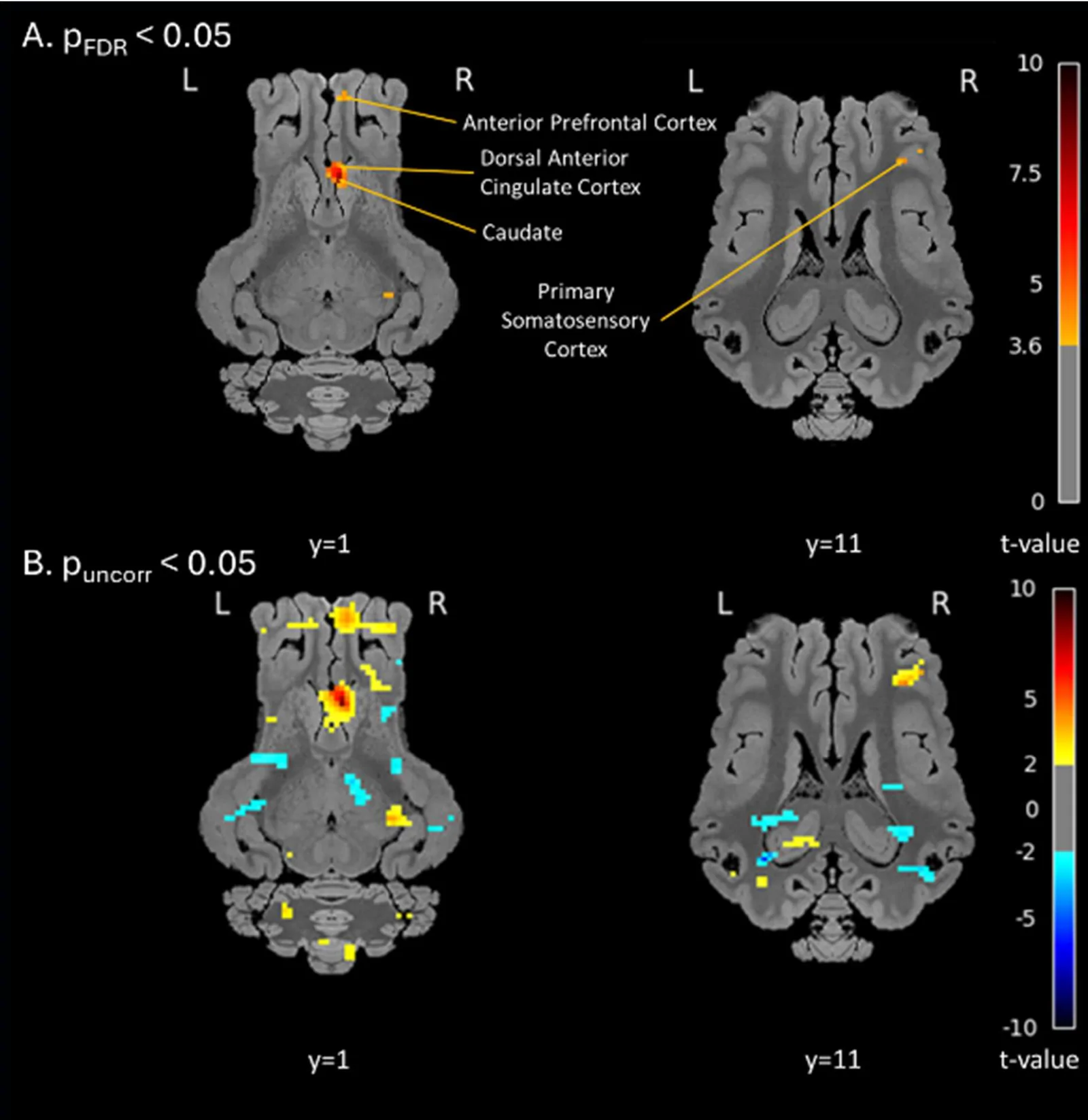
Brain activation maps in response to tactile stimulation (stroking) in control pigs (n = 8), naive to human stroking. (**A**) with FDR correction (*p*_FDR_ < 0.05; number of voxels per cluster k > 18) and (**B**) without correction (*p*_uncorr_ < 0.05). Horizontal maps of brain BOLD responses are represented at different dorsal–ventral levels (y) related to the posterior commissure (in mm). T-values are represented by the colour scale on the right. L = left; R = right.

Touch-elicited brain activation were detected in the right caudate nucleus, the right (two clusters) and left anterior prefrontal cortex, the right primary somatosensory cortex, and right dorsal anterior cingulate cortex as seen in the brain activation map of naive, control pigs (Fig. 1), which was confirmed by the corrected SVC-based statistics (Table 1).

**Table 1:** SVC-based statistics showing the brain responses to tactile stimulation (*i.e.* comparing stroking bouts to no stroking bouts) of pigs (n = 8) that were naive to human stroking (control). Related regions of interest (ROIs) with uncorrected *p*-value (P_uncorr_) that reached the criteria of *p* < 0.05 after Bonferroni correction (ROI number = 18).

	ROI	L/R	k	T	P_uncorr_	P_FDR_	Cohen’s d value	Coordinates [x, y, z]
Stroking > no stroking	Caudate nucleus	R	68	6.76	0.0003	0.20	1.875	[− 3, 23, 2]
	Primary Somatosensory Cortex	R	38	4.31	0.0025	0.86	1.195	[− 14, 27, 11]
	Anterior prefrontal cortex	L	89	4.53	0.0020	0.70	1.256	[9, 38, -1]
		R	23	5.53	0.0007	0.45	1.534	[− 1, 26, -3]
			86	4.69	0.0017	0.45	1.301	[− 3, 40, 0]
	Dorsal anterior cingulate cortex	R	18	6.2	0.0004	0.20	1.720	[− 2, 25, 2]

The test statistic (T) and the corrected *p*-value (False Discovery Rate, P_FDR_) are also presented, along with the laterality of the ROI (L = left, R = right), the size (k) and the coordinates of the cluster. Coordinates were calculated using the pig’s brain atlas^38^.

### Effect of positive human contact on the brain responses to tactile stimulation (stroking)

**Fig. 2:**
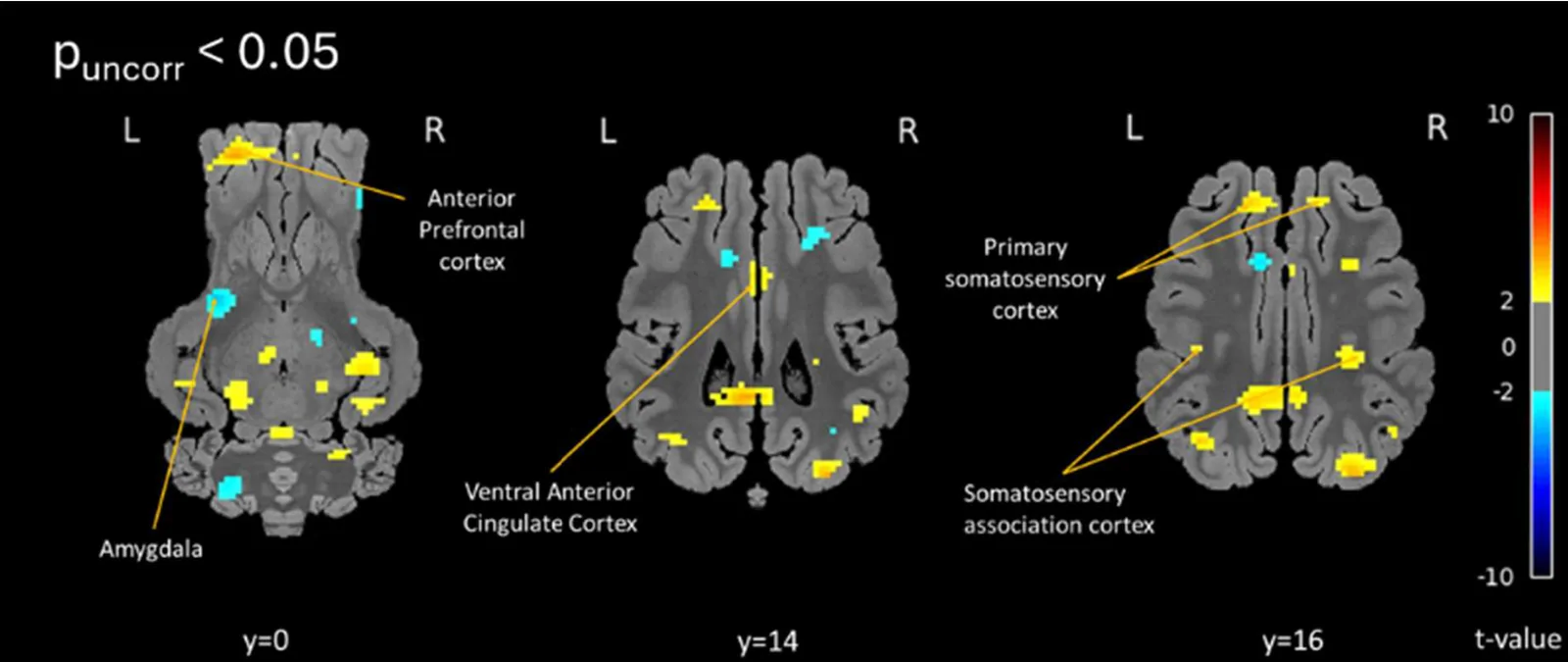
Contrast maps showing the peaks of activation in response to tactile stimulation in the brain of control pigs (in blue) and pigs which received positive human contacts (in yellow) (*p*_uncorr_ < 0.05; number of voxels per cluster k > 3). Horizontal maps of brain BOLD responses are represented at different dorsal–ventral levels (y) related to the posterior commissure (in mm). T-values are represented by the colour scale on the right. L = left; R = right.

The brain activation maps (Fig. 2) and the corrected SVC-based statistic (Table 2) showed a difference in brain responses to touch between POS pigs and CTL pigs. Only uncorrected activation maps are shown given that FDR correction could not provide any activated clusters. Control pigs showed greater activation in the right amygdala than POS pigs, whereas POS pigs showed greater activation in the right anterior prefrontal cortex, the right ventral anterior cingulate cortex, the left somatosensory association cortex and the right primary somatosensory cortex compared to CTL pigs. There was no difference between the two treatment groups in the brain activation of nucleus accumbens, caudate or putamen.

**Table 2:** SVC-based statistics comparing the brain response to tactile stimulation (stroking) of the control pigs (n = 8) and the pigs that had received regular positive human contacts (i.e. strokes; n = 7).

	ROI	L/R	k	T	P_uncorr_	P_FDR_	Cohen’s d value	Coordinates [x, y, z]
Control > Positive contacts	Amygdala	R	18	3.69	0.0014	0.07	1.023	[10, 13, − 1]
Positive contacts > Control	Anterior prefrontal cortex	R	113	4.14	0.0006	0.25	1.148	[9, 38, − 1]
	Primary Somatosensory Cortex	L	21	3.36	0.0026	0.40	0.932	[6, 17, 29]
	Somatosensory Association Cortex	R	42	3.35	0.0026	0.71	0.929	[− 12, 2, 17]
	Ventral Anterior Cingulate Cortex	R	3	3.61	0.0016	0.11	1.001	[2, − 4, 15]

Related regions of interest (ROIs) with uncorrected p-value (P_uncorr_) that reached the criteria of* p* < 0.05 after Bonferroni correction (ROI number = 18). The test statistic (T) and the corrected p-value (False Discovery Rate, P_FDR_) are also presented, along with the laterality of the ROI (L = left, R = right), the size (k) and the coordinates of the cluster. Coordinates were calculated using the pig’s brain atlas^38^

## Discussion

Touch by a human in the form of stroking elicited brain responses in pigs, even under general anaesthesia, with activation in the primary somatosensory cortex, but also in the caudate nucleus, anterior prefrontal cortex, and dorsal anterior cingulate cortex. These preliminary results suggest that the pigs perceived the tactile stimuli, and that gentle stroking by a human might be perceived as pleasant. In addition, pigs experienced with receiving regular positive human contacts showed greater activation in the prefrontal, ventral anterior cingulate, primary somatosensory and somatosensory association cortices, suggesting that stroking might be perceived as more pleasant, or at least that it mobilised integrative and higher-cognitive brain processes possibly involved in positive emotions. In contrast, control pigs showed greater activation in the amygdala, possibly due to the novelty of the situation for them. Despite large observed effect sizes (Cohen’s d = 0.93–1.15), these preliminary results must be taken with caution, given that statistical power was hindered (40–56%) by the limited sample size (planned: 10 pigs per treatment; final: CTL n = 8, POS n = 7) and consequent high inter-individual variability. Furthermore, the study design implies inferring mental states and processes from the activation of specific brain areas, i.e. reverse inference, which can be misleading because the activated brain areas could be involved in other mental processes than the ones considered^39^. Importantly, the stroking speed in the present study (i.e. about 20 cm/s) falls outside the optimal velocity range for activation of CT afferents (1–10 cm/s in humans and non-human primates). This limitation should be considered when interpreting the present findings in the context of affective-like touch processing. Specifically, the observed somatosensory activations may predominantly reflect discriminative tactile processing associated with higher-velocity stimulation, while any affective-like component should be interpreted cautiously.

The results of this study first confirmed that pigs under general anaesthesia perceived tactile stimuli, in the form of a gentle stroke along their spine. Other studies found that pigs under general anaesthesia could perceive olfactory^33–36^, gustatory^19,37^ or olfactogustatory (i.e. flavour)^40,41^ stimulations, as evidenced by differences in brain responses (BOLD with fMRI, blood brain perfusion with SPECT or glucose metabolism with PET) to the stimuli according to their previous experiences. In the present study, we compared the BOLD responses of the pig’s brain with and without stimulation, using CTL pigs to assess the naive response to the tactile stimulation. Brain activation differences were observed in some of our a priori brain regions of interest (ROI): The right caudate nucleus, the right and left anterior prefrontal cortex, the right primary somatosensory cortex, and right dorsal anterior cingulate cortex. This is in accordance to the human literature reporting that pleasant touch resulted in the activation of the caudate^21^, anterior cingulate cortex^22,24^ and prefrontal cortex^23^. Furthermore, in macaques, affective touch activated the insula and the anterior cingulate cortex^42^. Similar responses (i.e. activation in the right caudate, anterior prefrontal cortex, ventral anterior cingulate cortex) were observed in anaesthetised pigs that received paired oral and duodenal sucrose infusion compared to pigs that received only saline treatment, which was interpreted as an hedonic response^19^. Therefore, our results show brain activations that are consistent with neural processing associated with pleasant or affective tactile stimulation. However, contrary to our expectations based on the human literature reporting that stimulation of C-tactile afferents leads to an activation in the insula^43^, we found no difference in this area. This could be due to a failure to meet the optimal stroking velocity (3 cm/s in humans and non-human primates) and/or pressure for the stimulation of C-tactile afferents^44^. Indeed, greater activation of the insula was observed in the brain of lightly anaesthetized macaques when they were stroked at 3 cm/s compared to when they were stroked at 15 cm/s^42^. Moreover, although affective-like touch has been associated preferentially with activation of the insular cortex, particularly during low-velocity stimulation, the primary somatosensory cortex is more consistently linked to discriminative tactile processing, including the encoding of stimulus intensity, location, and movement velocity^45^. The somatosensory activations observed in the present study are therefore likely to reflect, at least in part, discriminative tactile processing associated with stroking on the back. However, because affective and discriminative components of touch processing are not fully separable at the neural level, these activations do not preclude a concurrent affective or salience-related component. While not matching the affective touch velocity, the tactile stimulation (stroking at approximately 20 cm/s) during the MRI scan was still not as fast and dynamic as the positive contacts provided during the treatment period, which the pigs seemed to enjoy based on their behavioural response (i.e. maintained contacts, pressed body against the hand, grunted and rounded their backs). Therefore, further studies could also investigate the perception of more rapid strokes and gentle scratching.

The second major finding of our study is the influence of long-term exposure to positive contacts with a human on the pigs’ brain responses to stroking. Pigs experienced to receiving regular human contacts showed activation differences in some of our pre-defined regions of interest compared to control pigs during the tactile stimulation. In agreement with our predictions, the BOLD response of POS pigs was higher in the left ventral anterior cingulate cortex, right anterior pre-frontal cortex, while CTL pigs had greater activation in the left amygdala. However, contrary to our predictions, there were no activation differences between groups in the putamen, caudate and insular cortex. Unexpectedly, there was a higher BOLD response in the primary somatosensory cortex and somatosensory association cortex in the POS pigs.

In humans, the perception of the pleasantness of a tactile stimulation seems to be influenced by the level of exposure to touch. Indeed, humans with low touch exposure (weekly or monthly) rated the pleasantness of being touched at the optimal velocity (3 cm/s) significantly lower than humans with a higher touch exposure (daily or weekly)^46^. For the other (suboptimal) velocities, both groups rated them as less pleasant and did not differ in their ratings^46^. This supports our finding that control pigs, although perceiving touch as pleasant, perceived it as less pleasant as pigs who received this contact three times weekly over nine weeks. However, the question remains whether pigs that received more allogrooming from their counterparts would react differently to stroking by a human. Allogrooming would result in a different sensory experience since it involves gentle nibbling and licking, thus leading to saliva deposits and calorific exchanges due to the variations of humidity on the skin, which is not the case with a human hand stroke. Further studies are warranted to elucidate the effect of intra-species touch on the perception of inter-species touch, and the extent of the inter-individuality variability in the perception of (pleasant) inter- and intra- species touch.

It has been proposed that the anterior cingulate cortex is the centre for action-output learning, implying its involvement in emotional process and memory of the link between rewards and actions^47^, but also in more complex processing such as personal meaning in humans^48^ (i.e. it is activated by memories of intrinsically motivated events^49^). The prefrontal cortex also plays a crucial role in processing, integrating and regulating experiences, including social interactions^50^. Specifically, the anterior prefrontal cortex has been hypothesised to play a role in recollection of contextual “internally generated” information (e.g. emotions) related to a past event (e.g. in our study, the positive contacts received in the home pen)^51^. In the context of the present study, those findings in humans could suggest that the tactile stimulation triggered neural mechanisms linked to information recollection and emotional regulation in the POS pigs, possibly showing that they associated the stroking stimulation with their past experience with the human in the home pen and that it may carry a personal meaning for those pigs. However, we could not find significant differences between POS and CTL pigs of activation in the caudate and putamen, which are also involved in reward processing^18^.

The differences in the activation in the amygdala were the most significant of all regions considered in the analysis. Our hypothesis was that we would observe a greater activation in the CTL pigs, for which the tactile stimulation from a human was an unknown stimulus, as a marker of surprise. Indeed, while both hippocampus and amygdala respond to novelty, only the amygdala was observed to have a greater BOLD response to unusual (*vs.* common) novel visual stimuli in humans^52^. Several studies found higher activation of the amygdala in response to both negative and positive visual stimulation^53^ relation to pleasant touch (e.g.^54^), and could therefore reflect increased salience rather than an (negative) emotional response. However, in the context of our study, it would seem more intuitive to interpret the greater activation in the amygdala in the CTL pigs as a marker of surprise or novelty, given that stroking was a novel type of experience for them, compared to the POS pigs experienced to interacting with the human in various ways including being stroked on the back.

Finally, the greater activation in the primary somatosensory cortex and in the somatosensory association cortex in the POS pigs were unexpected, as we assumed that its activation was purely due to the processing of the tactile stimulation. For instance, the neurons in the somatosensory cortex have a similar response to the social and non-social tactile stimuli in macaques^32^. However, it is also possible that the experience with positive tactile contacts participated in the development of a greater neuroplasticity in the POS pigs, resulting in more neural activation of the somatosensory cortex during the tactile stimulation^55^. For instance, humans became more sensitive to androstenone (*i.e.* lower detection threshold) through repetitive exposure, which was accompanied by an increased neural response (*i.e.* increased olfactory evoked potential measured by an electroencephalogram^56^). There is also some disagreement between studies on whether the somatosensory cortex and/or the anterior cingulate cortex encode the affective value of touch, as some studies found that pleasantness ratings of touch predicted activation in the anterior cingulate cortex and not in the somatosensory cortex (e.g. ^25^), but others found that the somatosensory cortex only was involved (e.g.^57^). Therefore, our study might bring further evidence that the somatosensory cortex may indeed be involved in the affective processing of touch.

Collectively, our preliminary results suggest that stroking is genuinely perceived as pleasant by pigs, and that experience might make the perception more salient. This is also supported by behavioural data (not shown). Indeed, on the last day of the experiment, after the MRI scans, the voluntary approach to the human was measured for all pigs and they were offered a 10 min positive contacts session in their home pen (i.e. similar to POS treatment). Despite taking longer to approach the human^58^, CTL pigs spent 36% of their time in contact with the human (POS: 55%, data not shown). However, future studies should use additional measures of emotional response (e.g. heart rate variability) to further assert our findings. A final word of caution should be added regarding the effects of the anaesthetic products on the BOLD responses. We avoided the use of inhalant anaesthetics (e.g. isoflurane, sevoflurane) because these agents are known to affect neurovascular coupling and cerebral blood flow, leading to reduced neural activity and impairing the quality and interpretation of fMRI^59,60^. Instead, medetomidine was administered to maintain general anaesthesia. Medetomidine is commonly used in fMRI studies due to its relatively stable effects on cerebral haemodynamics and neural activity, making it a suitable choice for preserving BOLD signal integrity during imaging^61^. Furthermore, medetomidine, especially at low doses (i.e. 0.1 mg/kg/h in mice and rats, 10 times the dosage used in our study), has been reported to preserve the functional connectivity of cortical and sub-cortical brain regions and the brain activity in response to stimulus-evoked (somatosensory) stimulations (e.g. electrical or ultra-sound stimulation)^62,63^. Although the effects of azaperone on BOLD response remain unstudied, ketamine has been reported not to significantly alter BOLD signals related to cognitive states^64^.

Overall, the preliminary results of this study are quite encouraging for using larger sample sizes in future studies and represent a princeps demonstration of the neurocognitive effects of positive tactile interactions with humans in pigs.

## Conclusions

Stroking by a human was perceived and processed by the brain of the pigs, even under general anaesthesia as shown by activation in the primary somatosensory cortex, caudate nucleus, anterior prefrontal cortex, and dorsal anterior cingulate cortex. Pigs experienced with receiving regular positive human contacts showed greater activation in the prefrontal, ventral anterior cingulate, primary somatosensory and somatosensory association cortices, which is in accordance with the processing of pleasant tactile stimulation. In contrast, control pigs showed greater activation in the amygdala, which might be linked to the novelty of the stimulation for them.

Those preliminary results not only confirmed the translation of previous findings between species, but they also provide novel knowledge regarding the perception of human gentle contacts by animals. Finally, this study contributed to further validating neuroimaging as a tool for the assessment of pigs’ emotions and welfare, especially in the context of human-animal interactions and physical contacts.

## Materials and methods

The material and methods of the study, as well as the rationale behind the selection of the ROIs, have been submitted as a pre-registration on OSF^65^ and can be found here: https://doi.org/10.17605/OSF.IO/HKR67.

The study is reported in accordance with the ARRIVE guidelines.

### Animals and housing

This study involved 24 female pigs (*Sus Scrofa domesticus;* Swiss Large White × Pietrain breed) used over two batches (6 pigs per group for each batch). Those pigs were born and sourced at the research farm of the veterinary medicine university of Vienna (“Hof Medau”). Only female pigs were used in this study as a mean to reduce variability and sample size, and because the overall project (that this study is part of) focused on female pigs. Pairs of female siblings were recruited at weaning, based on being healthy and of average weight. One sibling was allocated to the “positive human contacts” (POS) group and the other sibling was allocated to the “control” group (CTL). Pigs were housed in trios, making four trios (two per treatment) per batch. Twenty of these pigs (10 per treatment, all sibling pairs) were randomly selected and scanned in a 1.5T Magnetic Resonance scanner (Siemens Healthineers, Magnetom Espree 1.5 T; Siemens Medical Solutions) once at weaning (before the treatment started, at 5 weeks of age) and the same individuals at the end of the treatment (16 weeks of age).

At the end of the study, pigs were euthanised, as they could not be returned to the food chain, and no adoption solution (i.e. private ownership or sanctuary) could be found. The procedure was performed by a veterinarian. Pigs were first sedated by intramuscular injection of ketamine hydrochloride (Narketan®, 20 mg/kg Body Weight [BW]) and xylazine (Sedaxylan®, 2 mg/kg BW), and euthanized by intravenous injection of T61 MSD® at a dose of 4 to 6 ml/50 kg BW. Death was confirmed by the loss of the corneal reflex, cessation of respiration and heartbeat.

### Treatments

After the first MRI (Magnetic Resonance Imaging) session, treatments were applied to create a different experience regarding human-animal interactions. In the “positive human contacts” group (POS), pigs got opportunities to interact voluntarily with a human (OS) and received positive contacts (such as petting, stroking, scratching) for 10 min, twice a day (10:00–11:00 and 14:00–15:00), three days a week (Monday, Wednesday and Friday), over 9 consecutive weeks. The positive contacts could be applied on any part of the pig’s body, in an individual manner depending on what the pig seemed to enjoy, but mainly occurred on the neck (scratching behind the ears, stroking), back (stroking and scratching) and belly (rubbing). In the “control” group (CTL), pigs were only exposed to the presence of the same human, who was standing silently outside and in front of their home pen and avoiding eye contact, following the same schedule as for POS pigs.

### Anaesthesia protocol

Each pig underwent MRI scanning under general anaesthesia, performed by a certified veterinary anaesthetist (GM), using a refined protocol designed to optimize BOLD image acquisition^66^. The same anaesthetic protocol was used in all pigs and doses were adjusted according to age group and demeanour in order to minimize stress in the perianaesthetic period. Pigs were fasted for 4–8 h with access to water ad libitum until premedication. Each pig was sedated intramuscularly with ketamine (Narketan, 7–14 mg/kg BW) and azaperone (Stresnil®, 1–2 mg/kg BW) into the brachiocephalic muscle. Following injection, pigs were left undisturbed in a familiar, dark room (their home enclosure) for a variable period (5–20 min) until moderate sedation was achieved. Distance monitoring was used to avoid additional stress. If sedation was deemed insufficient, a second intramuscular injection of one-third to one-fourth of the initial doses was administered before transport to the preparation room. Pigs were kept in sternal recumbency on a padded table. A catheter (20G or 22G) was placed in the external auricular vein and secured. After preoxygenation for at least 5 min with 100% oxygen via mask, general anaesthesia was induced with ketamine (Narketan®, 20–30 mg/kg BW), azaperone (5 mg/kg BW) and atropine (0.04 mg/kg BW) intravenously. Pigs were orotracheally intubated with a cuffed endotracheal tube of appropriate size and the tube was secured. A second catheter (20G or 22G) was placed in the central auricular artery for invasive blood pressure monitoring. Eye lubricant was applied to prevent corneal drying. Pigs were then transported to the MRI suite, where the endotracheal tube was connected to a circle breathing system in 100% oxygen and to the anaesthetic machine. Mechanical ventilation was initiated, with tidal volume and respiratory rate adjusted to maintain end-tidal CO₂ between 40 and 45 mmHg. A continuous intravenous infusion of medetomidine (Domitor®, 0.018 mg/kg/h) was initiated. An isotonic crystalloid solution (Sterofundin ISO) was administered at a variable rate (5–10 ml/kg/h) throughout the procedure. Loss of consciousness and anaesthetic depth were evaluated clinically: absence of jaw tone and palpebral reflex, ventro-medial eye rotation, absence of spontaneous movements, tolerance of the endotracheal tube, and stable cardiovascular variables. Standard perioperative monitoring and care was applied and recorded: heart rate, electrocardiography, respiratory rate, pulse oximetry, capnography, spirometry, invasive blood pressure and rectal temperature were monitored using a multi-parameter monitor (HP CMS-2000 Anaesthesia monitor) throughout the procedure. Mean arterial blood pressure, arterial haemoglobin oxygen saturation and body temperature were maintained above 60 mmHg, 95% and 36 °C, respectively. At the end of the procedure, pigs were allowed to recover under close supervision. Atipamezole (Antisedan®, 0.05–0.1 mg/kg) was administered intramuscularly to antagonise the effects of medetomidine, facilitating recovery. Following tracheal extubation, oxygen was administered via facemask to support recovery. Intravenous crystalloid infusion was continued until the pigs were able to stand unaided. Once stable, each pig was returned to its enclosure and kept separated from the rest of the group until fully awake. The entire procedure, from sedation to end of general anaesthesia, lasted up to three hours. Recovery times varied among individuals, and was up to two hours.

### MRI scans procedure

**Fig. 3:**
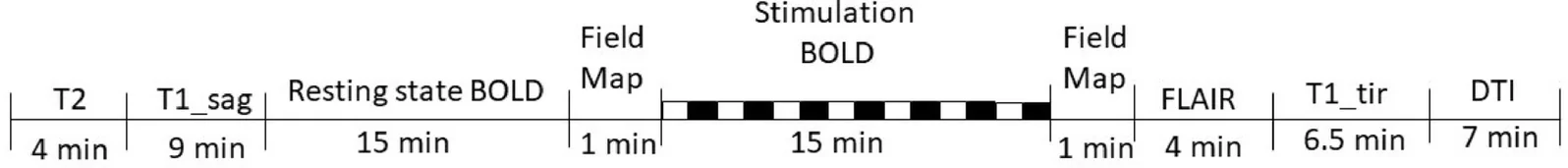
Schematic representation of the MRI session timeline. Within the “Stimulation BOLD” section, the black rectangles represent the 1-min periods of stroking stimulation (i.e. human stroking the pig’s back), and the white rectangles represent the 1-min periods without stimulation (i.e. human standing next to the pig in silence). T2: Transverse relaxation time, allows characterization of tissues by capturing differences in decay latency of spins’ aligned precession in the transverse plane. T1_sag: Longitudinal relaxation time performed in transversal view, allows characterization of tissues by capturing differences in the proton spins’ latency to relaxation from the sagittal plane toward the main longitudinal magnetic vector. T1_tir: Longitudinal relaxation time with turbo inversion recovery, similar to FLAIR. FLAIR: Fluid Attenuated Inversion Recovery, removes the signal from the cerebrospinal fluid and therefore allows to detect subtle changes at the periphery of the hemispheres and in the periventricular region close to CSF. DTI: Diffusion Tensor Imaging, allows mapping white matter’s nerve tracts. BOLD: Blood-Oxygen-Level-Dependent imaging, allows quantifying the variations of oxygen transported by haemoglobin in relation to neuronal activity.

For the need of the scanning procedure, the pig’s head was placed inside a human coil (knee coil at 5 weeks of age, head coil at 15 weeks of age). The scanning session lasted approximately 60 min and the MRI sequence included structural and functional imaging (Fig. 3). The images were acquired using the software Syngo.via (Siemens Healthcare GmbH, Germany). The details of the acquisition characteristics of each MRI sequence can be found in Table 3. The Blood-Oxygen-Level-Dependent (BOLD) functional imaging included a “resting state” (rsMRI), which aimed to assess the general neuronal activity and connectivity of the pigs’ brain, followed by a “stimulation” (BOLD fMRI), which aimed to assess pigs’ neuronal activity in response to stroking. The human involved in the weekly interactions (“familiar human”) performed the tactile stimulation during the fMRI, given that pigs may perceive and discriminate humans based on smell (and maintain a sense of smell even under general anaesthesia, e.g.^33^). During fMRI, the familiar human entered the MRI room and alternated between non-stimulation (i.e. just standing next to the scanned pig in silence) for one minute, and stroking the scanned pig on her back (soft stroke made by applying gently the full palm of the right hand, from shoulders to rumps along the spine) for one minute (1 stroke every 3 s, timing based on the pulses of the MR scanner) for a total of 20 repetitions. Strokes were delivered from the cranial to the caudal region of the back, hereby following the direction of hair growth. To avoid confounding effects, the interacting human was not involved in the handling of the pigs for the scanning procedure and remained in a separate room until the time of stimulation.

**Table 3:** Details of the acquisition characteristics of each MRI sequence.

	TR Repetition time (ms)	TE Echo time (ms)	FOV Field of view (mm × mm)	Matrix size	Number of slices
T2	5370	109	240 × 240	256 × 256	46
T1_sag	1170	5.58	230 × 230	256 × 208	88
T1_tir	7000	63	180 × 180	256 × 256	52
FLAIR	8500	86	180 × 180	512 × 448	25
DTI	3200	105	204 × 204	104 × 128	20
Field Map	460	4.78/9.54	192 × 192	64 × 64	36
BOLD (T2*)	3000	39	192 × 192	74 × 74	22

### Processing of images

For the present study, only the data (*i.e.* volumes representing BOLD signal in voxels) from the images acquired during the stimulation period (fMRI) were analysed, using SPM12. A segmented 3D atlas of the pig brain^38^ allowed to identify the regions of interest (ROIs), and to define the masks used for small-volume correction.

The selected regions of interest (ROIs) where those for which evidence of difference of activation due to social contact and/or pleasurable experience was found in the scientific literature, in pigs or other species (as cited in the introduction):

- Anterior prefrontal cortex
- Anterior cingulate cortex
- Insular cortex
- Dorsal striatum (putamen and caudate)
- Ventral striatum (nucleus accumbens)
- Amygdala
- Somatosensory cortex
- Somatosensory association cortex

Data analysis was adapted from Menneson et al.^34^ and performed with SPM12 (release 7771, Wellcome Dept. of Cognitive Neurology, London, United Kingdom) and using Nipype for pipelining^67^. Segmentation of the brain on the anatomical image was performed with a 3-tissue probability map co-registered with the Saikali atlas. Pre-processing of BOLD sequences consisted in slice-timing, realignment based on the first volume, co-registration between BOLD images and anatomical image, normalisation to the pig brain atlas using the same tissue probability map as for the segmentation. Then, images were smoothed with a Gaussian kernel of 4mm to improve the signal to noise ratio.

### Statistical analysis

The statistical unit was the individual pig. We used a whole-brain approach, but also applied small-volume correction (SVC) for our a priori regions of interest (ROI). Due to limitations related to the size of the pig’s brain and the effect of general anaesthesia on brain activity, we used a non-standard statistical analysis with regards to human statistical standards usually considering statistical significance at a cluster level with *p*-value < 0.05^34^. Using human statistical standard in large animal study is challenging because the MR setup and BOLD sequence analysis pipeline were developed for (awake) human brain, and BOLD signal variation is reduced in anaesthetised animals. For instance, BOLD signal variation detected in dogs under several anaesthesia regimes (not including medetomidine) is lower than signal variation in awake humans (about 1% signal variation versus 5%)^68^. Even if medetomidine shows minor impact on evoked vasoreactivity^69^ lower variation of BOLD signal in anaesthetised animals will consequently impair the detectability of human-standard statistics. For those reasons, a large consortium working with rats recently chose to show map for *p* < 0.05 without correction in order to reveal the full extent of the activation clusters and to avoid showing empty maps for low activation datasets^70^. In large animals, only intracranial stimulation has shown sufficient level of stimulation allowing the use of human statistical standard, as discussed in Coquery et al.^37^. Furthermore, the pig brain is much smaller than the human brain and the human MR setup is not perfectly well suited for pig acquisition. Furthermore, the bones of the pig skull are much thicker than those of humans, which ‘mechanically distances’ the brain from the antennas and thus impairing the signal to noise ratio (SNR).

For the whole brain approach we used a threshold set at *p* < 0.05 (with a False Discovery Rate (FDR) correction for the effect of touch analysis or without correction for the effect of positive contacts vs. control analysis) to produce the brain activation maps. FDR controls the proportion of false positives among all significant results, so it reduces the number of false positives compared to uncorrected p-values but it increases the false negative rate and some small effects can be missed. If FDR correction did not promote the detection of activated clusters, we used a threshold set at *p* < 0.05 without correction, consequently admitting that some activated cluster might be false positive. This strategy is currently suggested for non-human animal fMRI study^70^. For the SVC we used a *p*-value corrected for multiple ROIs comparisons with a Bonferroni correction and at a threshold of 0.05 (peak level). For this, and for each ROI, the uncorrected p-value was divided by the number of ROI (18) and was considered as statistically significant under Bonferroni correction if inferior at 0.05. Nine brain regions were analysed, corresponding to 18 ROIs bilaterally, thus the correction was applied for n = 18 ROIs. Corrected and uncorrected p-values are presented in the final results, since our study is exploratory and performed on a low number of animals^34,37^. Note that, because multiple of ROIs are examined with a relatively small sample size, the possibility of inflated false positive findings should be considered more carefully.

To investigate the differences in the brain responses to tactile stimulation (comparing images of naive pigs with and without stimulation), we ran a first-level analysis on each pig using a mass-univariate approach based on General Linear Model (GLM). Betas estimators of the model were then used to compute the different contrasts. Those contrasts fed the second-level analysis, also referred as “Random-Effects” or “Mixed-Effects” analysis as it considers both within- and between-subject variance. For this hypothesis, a one-sample t-test was used to compare brain responses to tactile stimulation and non-stimulation bouts in CTL pigs (n = 8) at the second time point (“after” treatment application). The random effects of pig, pen and replicate were accounted for in the models.

To investigate the difference due to exposure to positive human contacts, we ran a two-sample t-test-factor ANOVA, where we compared pigs from the two experimental treatments (CTL n = 8; POS n = 7) at the second session (“after” treatment application). The random effects of pig, pen and replicate were accounted for in the models. We looked at activation differences using both a whole-brain and a SVC approach using the same procedure as above. Additionally, the Cohen’s d-value was added in order to report effect size.

Some data were excluded from the analysis, due to the following reasons:

−Whole sequence, problems due to acquisition:

- misplacing of the pig (n = 2, pigs 8485 and 9391)
- intensity problem (n = 1; pig 8599)
- artefact in the sequence in the prefrontal region (n = 2; pigs 8514 and 9349)

−Volumes in sequence (n = 122 volumes excluded from 30 sequences; range: 0–19 volumes excluded per sequence):

- volumes with too much displacement (rotation and/or translation) compared to the preceding ones
- volumes with too much change in intensity compared to the preceding ones

These five exclusions were roughly balanced across treatment groups (2 in CTL, 3 in POS).

## Data Availability

All data generated or analysed during this study are included in this article. The contrast maps, ROI-wise statistics and summary tables of the results can be found on Open Science Framework repository: https://osf.io/b7jad/?view_only=e7d2eaf3a64646d08655590b18421640. A pre-print of this manuscript can be found on BioRxiv (deposited on 26th August 2025): https://doi.org/10.1101/2025.08.21.671579v1. Further inquiries can be directed to the corresponding authors.

## References

[1] Hemsworth, P. H. & Coleman, G. J. Human-animal interactions and animal productivity and welfare. In *Human-Livestock Interactions: The Stockperson and the Productivity and Welfare of Intensively Farmed Animals* (eds Hemsworth, P. H. & Coleman, G. J.) 47–83 (CABI, 2011). 10.1079/9781845936730.0047.

[2] Mota-Rojas, D. et al. Effects of human-animal relationship on animal productivity and welfare. *J. Anim. Behav. Biometeorol.* **8**, 196–205 (2020).

[3] Rushen, J., Taylor, A. A. & De Passille, A. M. Domestic animals’ fear of humans and its effects on their welfare. *Appl. Anim. Behav. Sci.* **65**, 285–303 (1999).

[4] Rault, J. L., Waiblinger, S., Boivin, X. & Hemsworth, P. The power of a positive human-animal relationship for animal welfare. *Front. Vet. Sci.* **7**, 1–13 (2020). 10.3389/fvets.2020.590867 PMC7680732 33240961

[5] Lucas, M. E. et al. Early human contact and housing for pigs – Part 2: Resilience to routine husbandry practices. *Animal* **18**, 101165 (2024). 10.1016/j.animal.2024.101165 38776694

[6] Lucas, M. E. et al. Early human contact and housing for pigs—Part 1: Responses to humans, novelty and isolation. *Animal* **18**, 101164 (2024). 10.1016/j.animal.2024.101164 38761440

[7] Hemsworth, P. H. Human-animal interactions in livestock production. *Appl. Anim. Behav. Sci.* **81**, 185–198 (2003).

[8] Brajon, S., Laforest, J. P., Schmitt, O. & Devillers, N. The way humans behave modulates the emotional state of piglets. *PLoS ONE* **10**, 1–17 (2015). 10.1371/journal.pone.0133408 PMC4526664 26244335

[9] Rygula, R., Pluta, H. & Popik, P. Laughing rats are optimistic. *PLoS ONE*10.1371/journal.pone.0051959 (2012). PMC3530570 23300582

[10] Camerlink, I., Scheck, K., Cadman, T. & Rault, J.-L. Lying in spatial proximity and active social behaviours capture different information when analysed at group level in indoor-housed pigs. *Appl. Anim. Behav. Sci.* **246**, 105540 (2022).

[11] Clouard, C. et al. Evidence of stable preferential affiliative relationships in the domestic pig. *Anim. Behav.* **213**, 95–105 (2024).

[12] Tallet, C., Brajon, S., Devillers, N. & Lensink, J. Pig-human interactions: Creating a positive perception of humans to ensure pig welfare. In *Advances in Pig Welfare* 381–398 (Elsevier Inc., 2018). 10.1016/B978-0-08-101012-9.00008-3.

[13] Truong, S., Schmitt, O. & Rault, J.-L. On your terms or mine: Pigs’ response to imposed gentle tactile contact vs. free form interaction with a familiar human. *Sci. Rep.* **14**, 25249 (2024). 10.1038/s41598-024-76451-5 PMC11502703 39448780

[14] Ishiyama, S. & Brecht, M. Neural correlates of ticklishness in the rat somatosensory cortex. *Science* **354**, 757–760 (2016). 10.1126/science.aah5114 27846607

[15] Rault, J. L., van den Munkhof, M. & Buisman-Pijlman, F. T. A. Oxytocin as an indicator of psychological and social well-being in domesticated animals: a critical review. *Front. Psychol.* **8**, (2017). 10.3389/fpsyg.2017.01521 PMC5601408 28955264

[16] Bertenshaw, C. & Rowlinson, P. Exploring heifers’ perception of ‘positive’ treatment through their motivation to pursue a retreated human. *Anim. Welf.* **17**, 313–319 (2008).

[17] Rault, J. L. et al. Gentle abdominal stroking (‘belly rubbing’) of pigs by a human reduces EEG total power and increases EEG frequencies. *Behav. Brain Res.* **374**, 111892 (2019). 10.1016/j.bbr.2019.04.006 30959126

[18] Berridge, K. C. & Kringelbach, M. L. Affective neuroscience of pleasure: Reward in humans and animals. *Psychopharmacology* **199**, 457–480 (2008). 10.1007/s00213-008-1099-6 PMC3004012 18311558

[19] Clouard, C., Meunier-Salaün, M. C., Meurice, P., Malbert, C. H. & Val-Laillet, D. Combined compared to dissociated oral and intestinal sucrose stimuli induce different brain hedonic processes. *Front. Psychol.* **5**, 1–12 (2014). 10.3389/fpsyg.2014.00861 PMC4124794 25147536

[20] Klein, J. T. & Platt, M. L. Social information signaling by neurons in primate striatum. *Curr. Biol.* **23**, 691–696 (2013). 10.1016/j.cub.2013.03.022 PMC3654103 23562270

[21] Sailer, U. et al. Temporal dynamics of brain activation during 40 minutes of pleasant touch. *Neuroimage* **139**, 360–367 (2016). 10.1016/j.neuroimage.2016.06.031 27338514

[22] Lindgren, L. et al. Pleasant human touch is represented in pregenual anterior cingulate cortex. *Neuroimage* **59**, 3427–3432 (2012). 10.1016/j.neuroimage.2011.11.013 22100768

[23] Kida, T. & Shinohara, K. Gentle touch activates the anterior prefrontal cortex: An NIRS study. *Neurosci. Res.* **76**, 76–82 (2013). 10.1016/j.neures.2013.03.006 23538146

[24] Rolls, E. T. Representations of pleasant and painful touch in the human orbitofrontal and cingulate cortices. *Cereb. Cortex* **13**, 308–317 (2003). 10.1093/cercor/13.3.308 12571120

[25] Case, L. K. et al. Encoding of touch intensity but not pleasantness in human primary somatosensory cortex. *J. Neurosci.* **36**, 5850–5860 (2016). 10.1523/JNEUROSCI.1130-15.2016 PMC4879201 27225773

[26] Kirsch, L. P. et al. Damage to the right insula disrupts the perception of affective touch. *Elife* **9**, 1–17 (2020). 10.7554/eLife.47895 PMC7043887 31975686

[27] Morrison, I., Björnsdotter, M. & Olausson, H. Vicarious responses to social touch in posterior insular cortex are tuned to pleasant caressing speeds. *J. Neurosci.* **31**, 9554–9562 (2011). 10.1523/JNEUROSCI.0397-11.2011 PMC6623148 21715620

[28] Gordon, I. et al. Brain mechanisms for processing affective touch. *Hum. Brain Mapp.* **34**, 914–922 (2013). 10.1002/hbm.21480 PMC6869848 22125232

[29] Obreja, O. & Schmelz, M. Single-fiber recordings of unmyelinated afferents in pig. *Neurosci. Lett.* **470**, 175–179 (2010). 10.1016/j.neulet.2009.10.006 19818831

[30] Lind, N. M. et al. The use of pigs in neuroscience: Modeling brain disorders. *Neurosci. Biobehav. Rev.* **31**, 728–751 (2007). 10.1016/j.neubiorev.2007.02.003 17445892

[31] Marti, R. et al. Effects of contact with a dog on prefrontal brain activity: a controlled trial. *PLoS ONE* **17**, 1–19 (2022). 10.1371/journal.pone.0274833 PMC9534402 36197880

[32] Martin, A. B. et al. A context-dependent switch from sensing to feeling in the primate amygdala. *Cell Rep.* **42**, 112056 (2023). 10.1016/j.celrep.2023.112056 PMC10430631 36724071

[33] Briard, E. et al. Exposure to a sensory functional ingredient in the pig model modulates the blood-oxygen-level dependent brain responses to food odor and acute stress during pharmacological MRI in the frontostriatal and limbic circuits. *Front. Nutr.* **10**, 1123162 (2023). 10.3389/fnut.2023.1123162 PMC10012862 36925960

[34] Coquery, N. et al. FMRI-based brain responses to olfactory stimulation with two putatively orexigenic functional food ingredients at two different concentrations in the pig model. *J. Food Sci.* **84**, 2666–2673 (2019). 10.1111/1750-3841.14772 31441517

[35] Menneson, S. et al*.* Validation of a psychosocial chronic stress model in the pig using a multidisciplinary approach at the gut-brain and behavior levels. *Front. Behav. Neurosci.* **13**, (2019). 10.3389/fnbeh.2019.00161 PMC6646532 31379533

[36] Menneson, S. et al. Regular exposure to a citrus-based sensory functional food ingredient alleviates the BOLD brain responses to acute pharmacological stress in a pig model of psychosocial chronic stress. *PLoS ONE* **15**, e0243893 (2020). 10.1371/journal.pone.0243893 PMC7769264 33370353

[37] Coquery, N. et al. FMRI-based brain responses to quinine and sucrose gustatory stimulation for nutrition research in the minipig model: A proof-of-concept study. *Front. Behav. Neurosci.* **12**, 1–9 (2018). 10.3389/fnbeh.2018.00151 PMC6094987 30140206

[38] Saikali, S. et al. A three-dimensional digital segmented and deformable brain atlas of the domestic pig. *J. Neurosci. Methods* **192**, 102–109 (2010). 10.1016/j.jneumeth.2010.07.041 20692291

[39] Poldrack, R. A. Inferring mental states from neuroimaging data: From reverse inference to large-scale decoding. *Neuron* **72**, 692–697 (2011). 10.1016/j.neuron.2011.11.001 PMC3240863 22153367

[40] Gaultier, A., Meunier-Salaün, M. C., Malbert, C. H. & Val-Laillet, D. Flavour exposures after conditioned aversion or preference trigger different brain processes in anaesthetised pigs. *Eur. J. Neurosci.* **34**, 1500–1511 (2011). 10.1111/j.1460-9568.2011.07848.x 22004412

[41] Val-Laillet, D., Meurice, P. & Clouard, C. Familiarity to a feed additive modulates its effects on brain responses in reward and memory regions in the pig model. *PLoS ONE* **11**, e0162660 (2016). 10.1371/journal.pone.0162660 PMC5017780 27610625

[42] Charbonneau, J. A. et al. Evolutionarily conserved neural responses to affective touch in monkeys transcend consciousness and change with age. *Proc. Natl. Acad. Sci. USA.* **121**, e2322157121 (2024). 10.1073/pnas.2322157121 PMC11067024 38648473

[43] Olausson, H. et al. Unmyelinated tactile afferents signal touch and project to insular cortex. *Nat. Neurosci.* **5**, 900–904 (2002). 10.1038/nn896 12145636

[44] Schirmer, A., Croy, I. & Ackerley, R. What are C-tactile afferents and how do they relate to “affective touch”?. *Neurosci. Biobehav. Rev.* **151**, 105236 (2023). 10.1016/j.neubiorev.2023.105236 37196923

[45] Morrison, I. ALE meta-analysis reveals dissociable networks for affective and discriminative aspects of touch. *Hum. Brain Mapp.* **37**, 1308–1320 (2016). 10.1002/hbm.23103 PMC5066805 26873519

[46] Sailer, U. & Ackerley, R. Exposure shapes the perception of affective touch. *Dev. Cogn. Neurosci.* **35**, 109–114 (2019). 10.1016/j.dcn.2017.07.008 PMC6969125 28818429

[47] Rolls, E. T. The cingulate cortex and limbic systems for emotion, action, and memory. *Brain Struct. Funct.* **224**, 3001–3018 (2019). 10.1007/s00429-019-01945-2 PMC6875144 31451898

[48] Matsunaga, M. et al. Structural and functional associations of the rostral anterior cingulate cortex with subjective happiness. *Neuroimage* **134**, 132–141 (2016). 10.1016/j.neuroimage.2016.04.020 27085503

[49] Lee, W. & Reeve, J. Remembering pleasure and personal meaning from episodes of intrinsic motivation: An fMRI study. *Motiv. Emot.* **44**, 810–818 (2020).

[50] Mohapatra, A. N. & Wagner, S. The role of the prefrontal cortex in social interactions of animal models and the implications for autism spectrum disorder. *Front. Psychiatry* **14**, 1–12 (2023). 10.3389/fpsyt.2023.1205199 PMC10318347 37409155

[51] Simons, J. S., Owen, A. M., Fletcher, P. C. & Burgess, P. W. Anterior prefrontal cortex and the recollection of contextual information. *Neuropsychologia* **43**, 1774–1783 (2005). 10.1016/j.neuropsychologia.2005.02.004 16154453

[52] Blackford, J. U., Buckholtz, J. W., Avery, S. N. & Zald, D. H. A unique role for the human amygdala in novelty detection. *Neuroimage* **50**, 1188–1193 (2010). 10.1016/j.neuroimage.2009.12.083 PMC2830341 20045069

[53] Liberzon, I., Phan, K. L., Decker, L. R. & Taylor, S. F. Extended amygdala and emotional salience: A PET activation study of positive and negative affect. *Neuropsychopharmacology* **28**, 726–733 (2003). 10.1038/sj.npp.1300113 12655318

[54] Chen, Y. et al. Oxytocin increases the pleasantness of affective touch and orbitofrontal cortex activity independent of valence. *Eur. Neuropsychopharmacol.* **39**, 99–110 (2020). 10.1016/j.euroneuro.2020.08.003 32861545

[55] Nithianantharajah, J. & Hannan, A. J. Enriched environments, experience-dependent plasticity and disorders of the nervous system. *Nat. Rev. Neurosci.* **7**, 697–709 (2006). 10.1038/nrn1970 16924259

[56] Wang, L., Chen, L. & Jacob, T. Evidence for peripheral plasticity in human odour response. *J. Physiol.* **554**, 236–244 (2004). 10.1113/jphysiol.2003.054726 PMC1664749 14678505

[57] Gazzola, V. *et al.* Primary somatosensory cortex discriminates affective significance in social touch. *Proc. Natl. Acad. Sci.* **109**, (2012). 10.1073/pnas.1113211109 PMC3382530 22665808

[58] Schmitt, O. et al. Regular positive human contacts do not improve pigs’ response to a lipopolysaccharide immune challenge. *NeuroImmunoModulation* **32**, 83–93 (2025). 10.1159/000544748 40024244

[59] Weber, R., Ramos-Cabrer, P., Wiedermann, D., Van Camp, N. & Hoehn, M. A fully noninvasive and robust experimental protocol for longitudinal fMRI studies in the rat. *Neuroimage* **29**, 1303–1310 (2006). 10.1016/j.neuroimage.2005.08.028 16223588

[60] Williams, K. A. et al. Comparison of α-chloralose, medetomidine and isoflurane anesthesia for functional connectivity mapping in the rat. *Magn. Reson. Imaging* **28**, 995–1003 (2010). 10.1016/j.mri.2010.03.007 PMC3740561 20456892

[61] Weber, R., Ramos-Cabrer, P., Wiedermann, D. & Hoehn, M. A longitudinal and totally noninvasive fMRI protocol in rats. *J. Cereb. Blood Flow Metab.* **25**, S361–S361 (2005).

[62] Lee, D., Kim, D. Y., To, X. V., Nasrallah, F. A. & Lee, H.-K. High-dose medetomidine increases functional connectivity in the fear-related regions after electrical stimulation. *Sci. Rep.* **15**, 33264 (2025). 10.1038/s41598-025-18556-z PMC12475416 41006490

[63] Sirmpilatze, N., Baudewig, J. & Boretius, S. Temporal stability of fMRI in medetomidine-anesthetized rats. *Sci. Rep.* **9**, 16673 (2019). 10.1038/s41598-019-53144-y PMC6853937 31723186

[64] Abel, K. M. et al. Ketamine and fMRI BOLD signal: Distinguishing between effects mediated by change in blood flow versus change in cognitive state. *Hum. Brain Mapp.* **18**, 135–145 (2003). 10.1002/hbm.10064 PMC6871893 12518293

[65] Schmitt, O. et al. An fMRI exploratory study of the effects of long-term positive human interactions on the pig’s brain resting state and BOLD responses to stroking. Preprint at 10.17605/OSF.IO/HKR67 (2024).

[66] Benn, R. A. That’ll do Pig, That’ll do: A common space approach to comparative neuroimaging extends the translational niche of the pig in neuroscience. (Universidad Autonoma de Madrid, 2021).

[67] Gorgolewski, K. *et al.* Nipype: A flexible, lightweight and extensible neuroimaging data processing framework in Python. *Front. Neuroinformatics* **5**, (2011). 10.3389/fninf.2011.00013 PMC3159964 21897815

[68] Willis, C. K. R. et al. Functional MRI as a tool to assess vision in dogs: the optimal anesthetic. *Vet. Ophthalmol.* **4**, 243–253 (2001). 10.1046/j.1463-5216.2001.00183.x 11906659

[69] Munting, L. P. et al. Influence of different isoflurane anesthesia protocols on murine cerebral hemodynamics measured with pseudo-continuous arterial spin labeling. *NMR Biomed.* **32**, e4105 (2019). 10.1002/nbm.4105 PMC6772066 31172591

[70] Galteau, M. E. et al. Activation mapping in multi-center retrospective rat sensory-evoked functional MRI datasets using a unified pipeline. *Imaging Neurosci.* **3**, IMAG.a.157 (2025). 10.1162/IMAG.a.157 PMC12455057 40995458

